# Supplementary figures and images for: An essential, kinetoplastid-specific GDP-Fuc: β-D-Gal α-1,2-fucosyltransferase is located in the mitochondrion of Trypanosoma brucei
Source: eLife. 2021 Aug 19;10:e70272. doi: 10.7554/eLife.70272 (PMC8439653; doi:10.7554/eLife.70272)

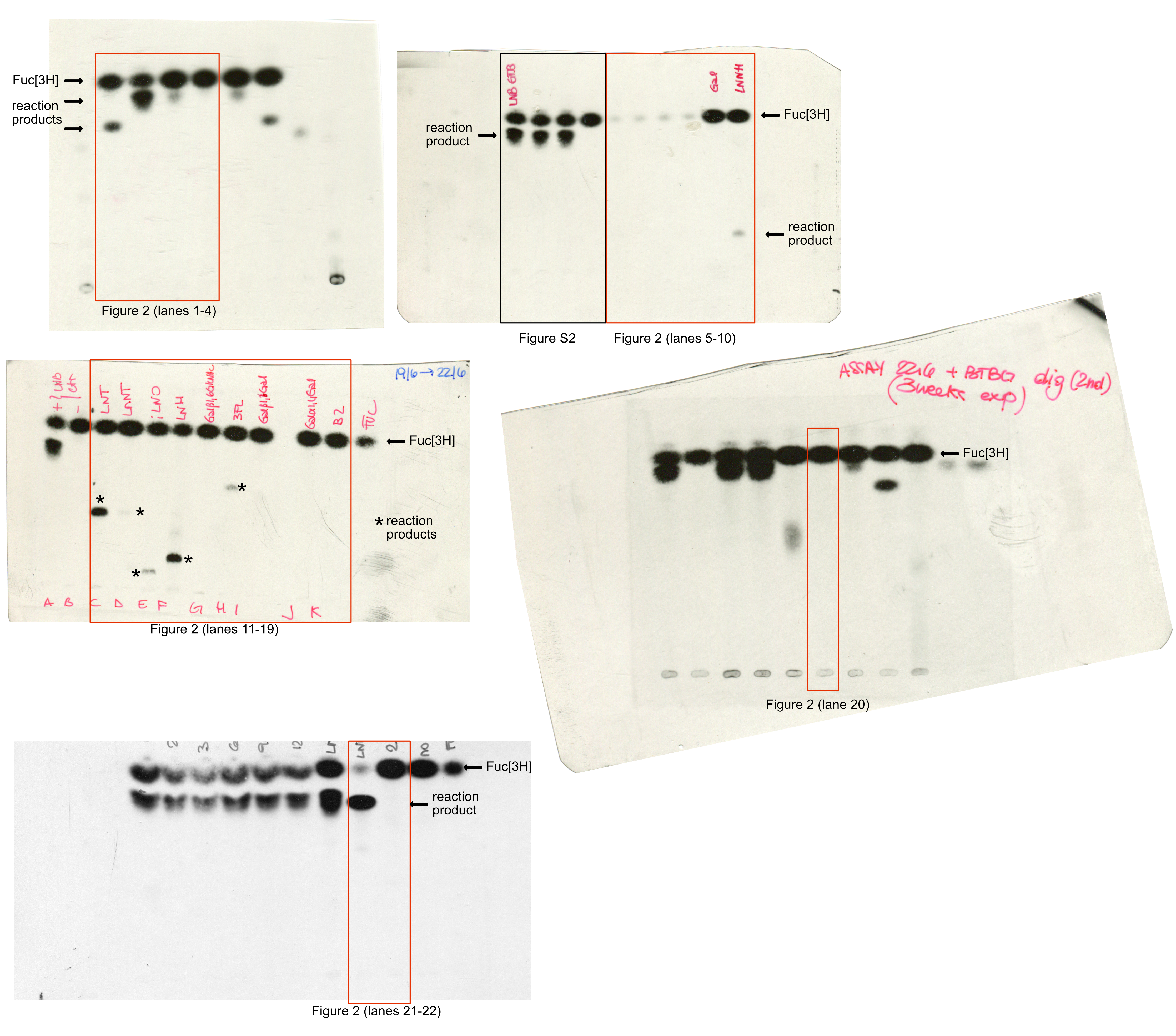

Supplement: Source data 1. [file elife-70272-supp3.zip › Source data 1/Figure 2 and figure 2 - figure supplement 2 allsource data.tiff]

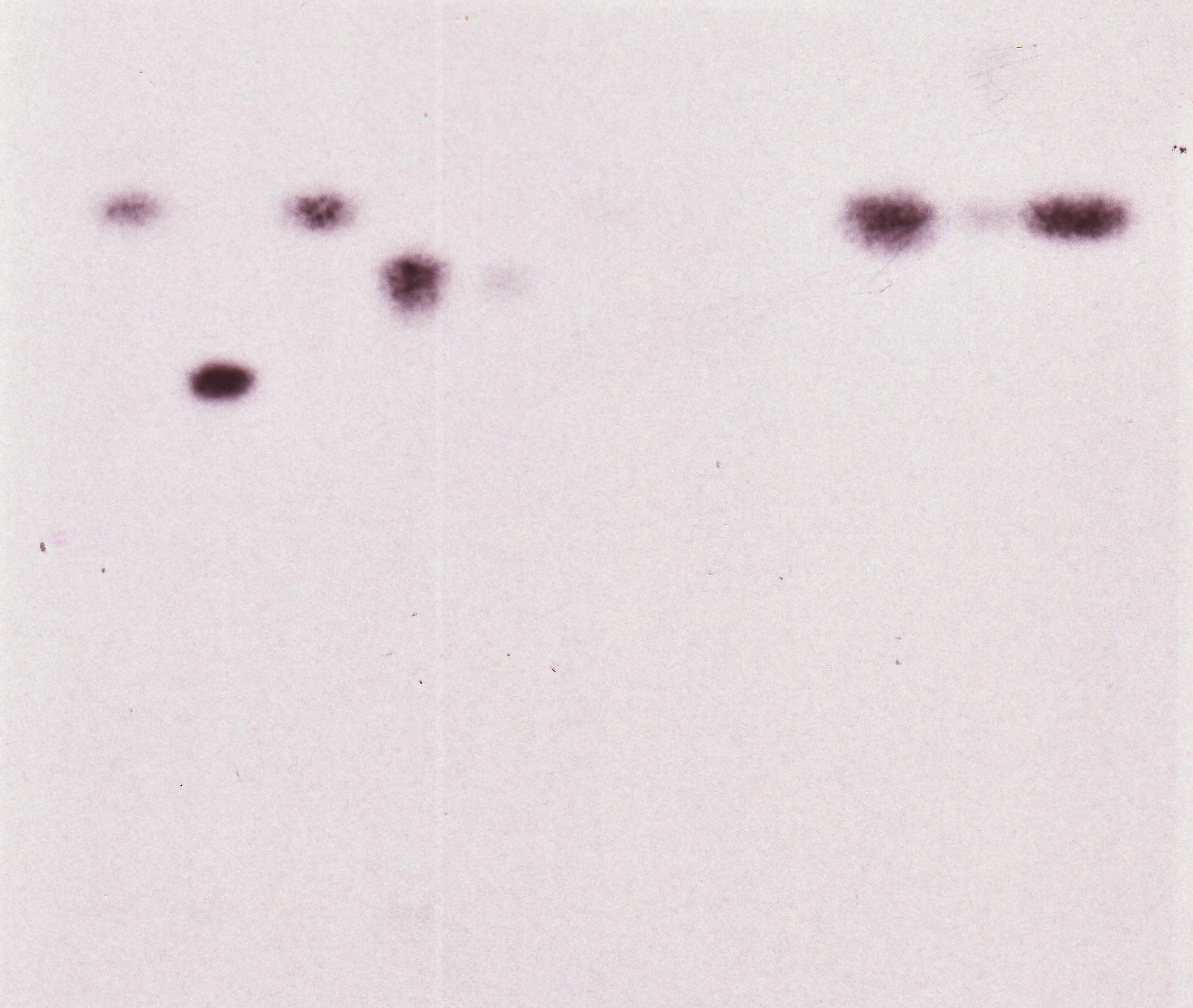

Supplement: Source data 1. [file elife-70272-supp3.zip › Source data 1/Figure 3 - figure supplement 1 panels A and B source data.tif]

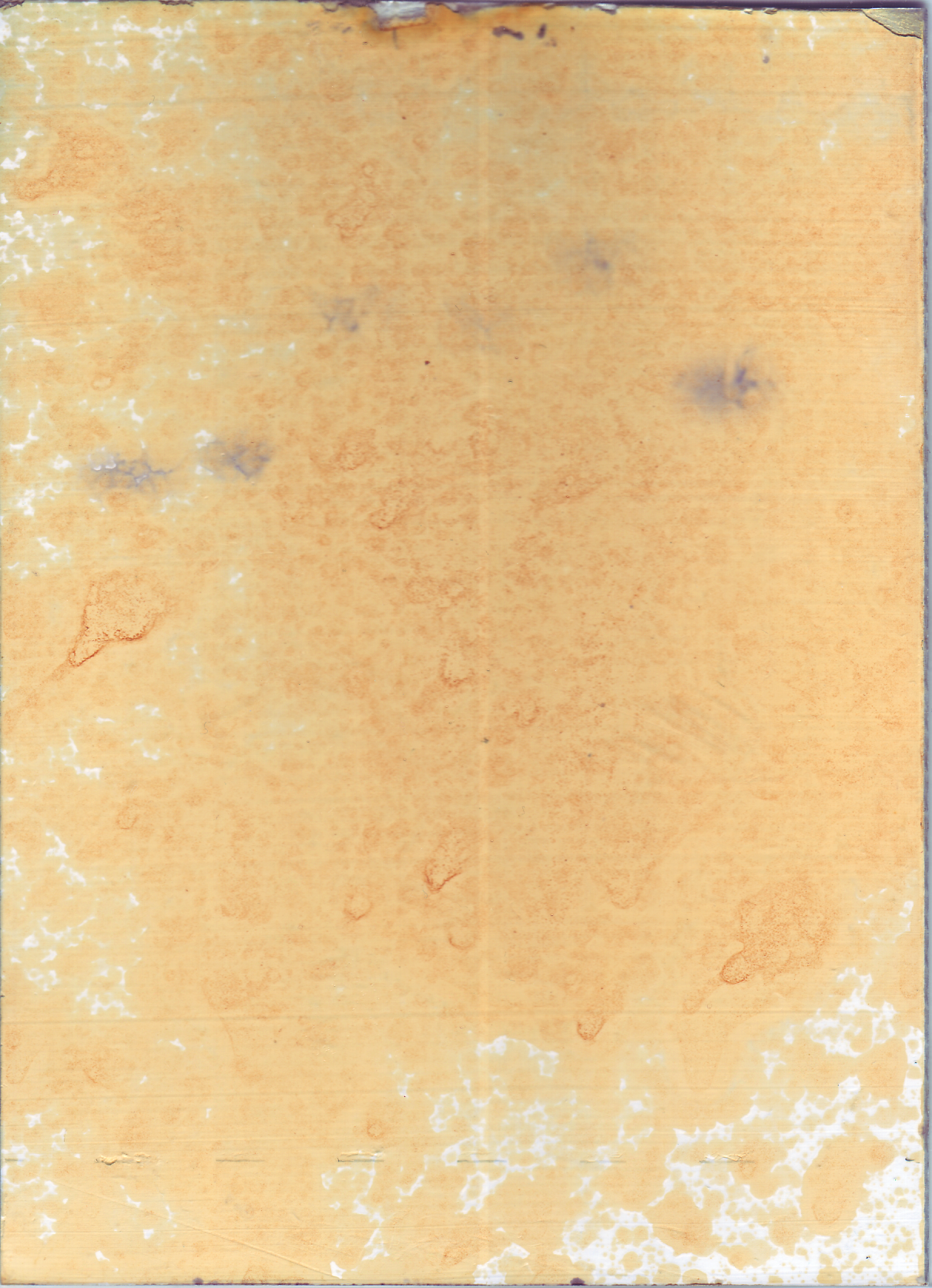

Supplement: Source data 1. [file elife-70272-supp3.zip › Source data 1/Figure 3 - figure supplement 1 panel C source data.tif]

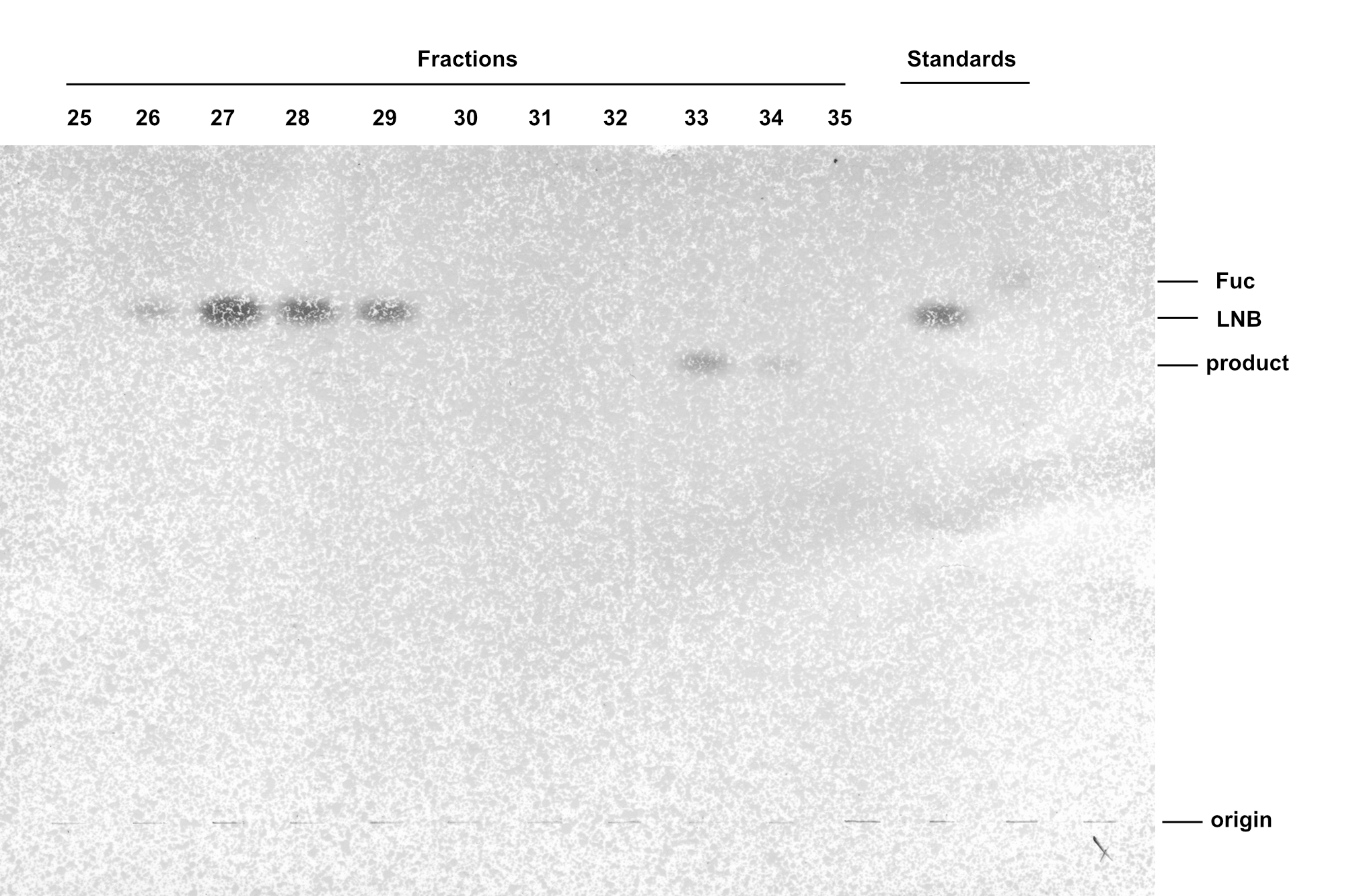

Supplement: Source data 1. [file elife-70272-supp3.zip › Source data 1/Figure 3- figure supplement 2 source data 2.tif]

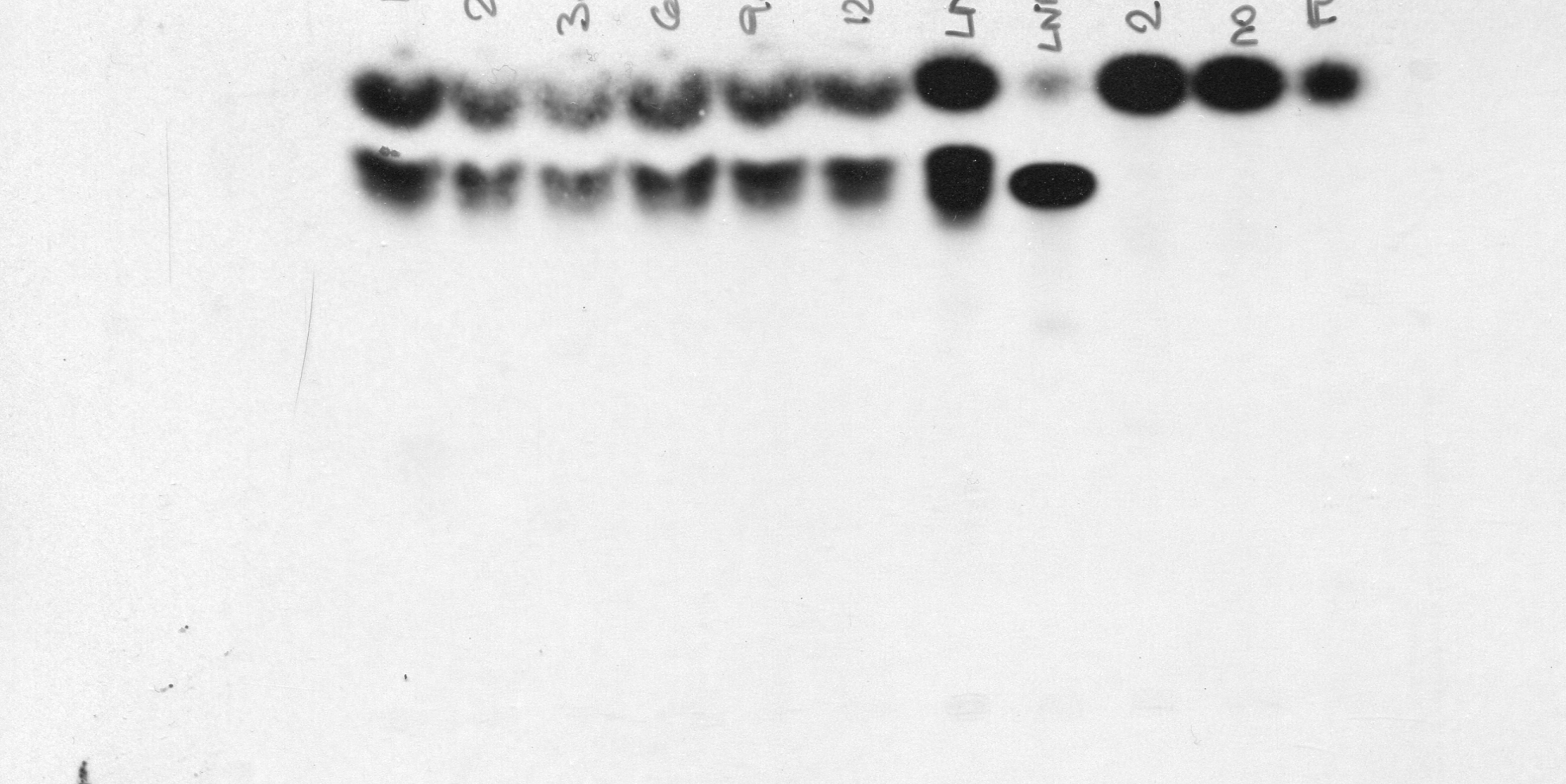

Supplement: Source data 1. [file elife-70272-supp3.zip › Source data 1/Figure 2 lanes 21-23 source data.tif]

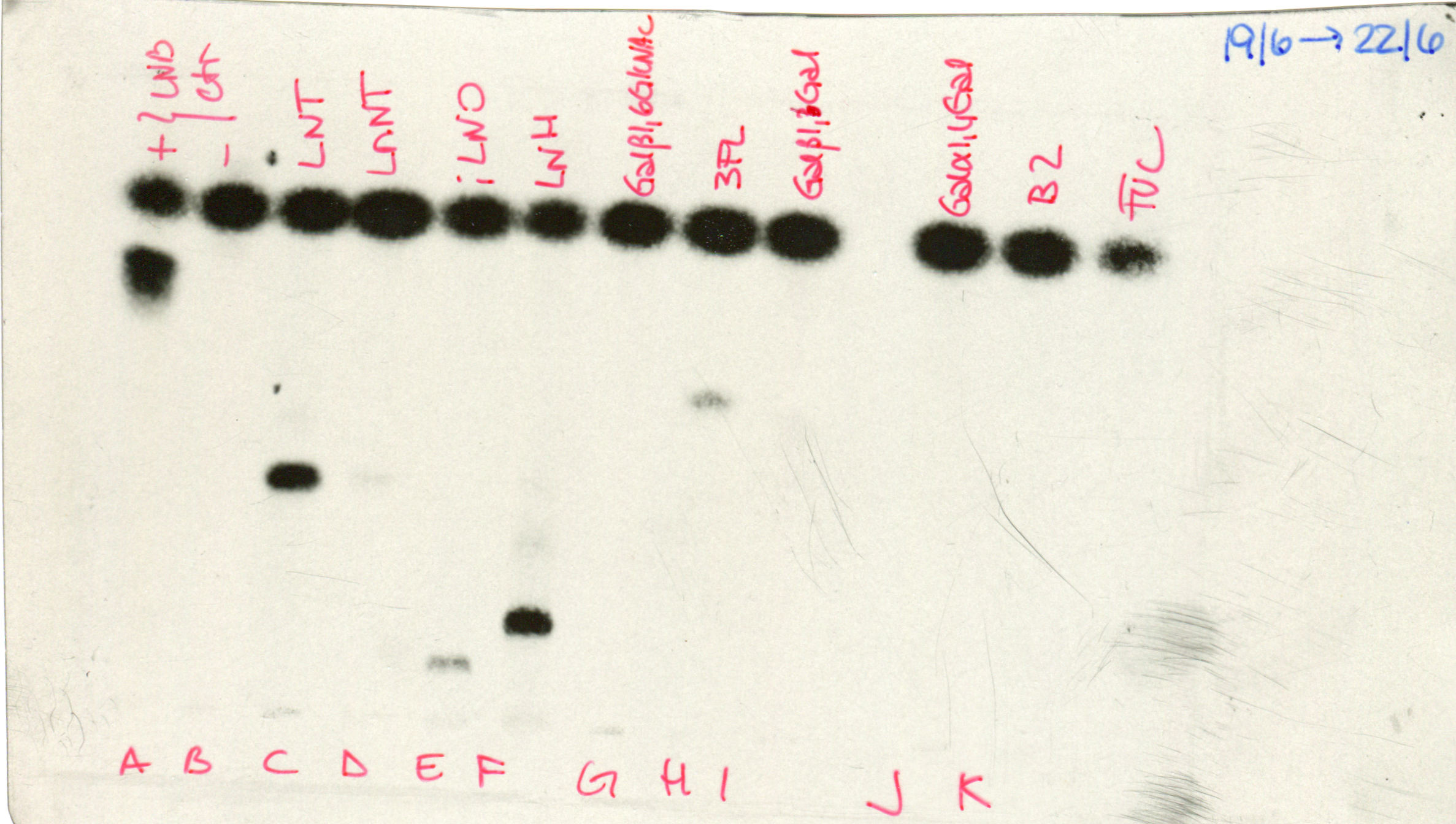

Supplement: Source data 1. [file elife-70272-supp3.zip › Source data 1/Figure 2 lanes 11-19 source data.tif]

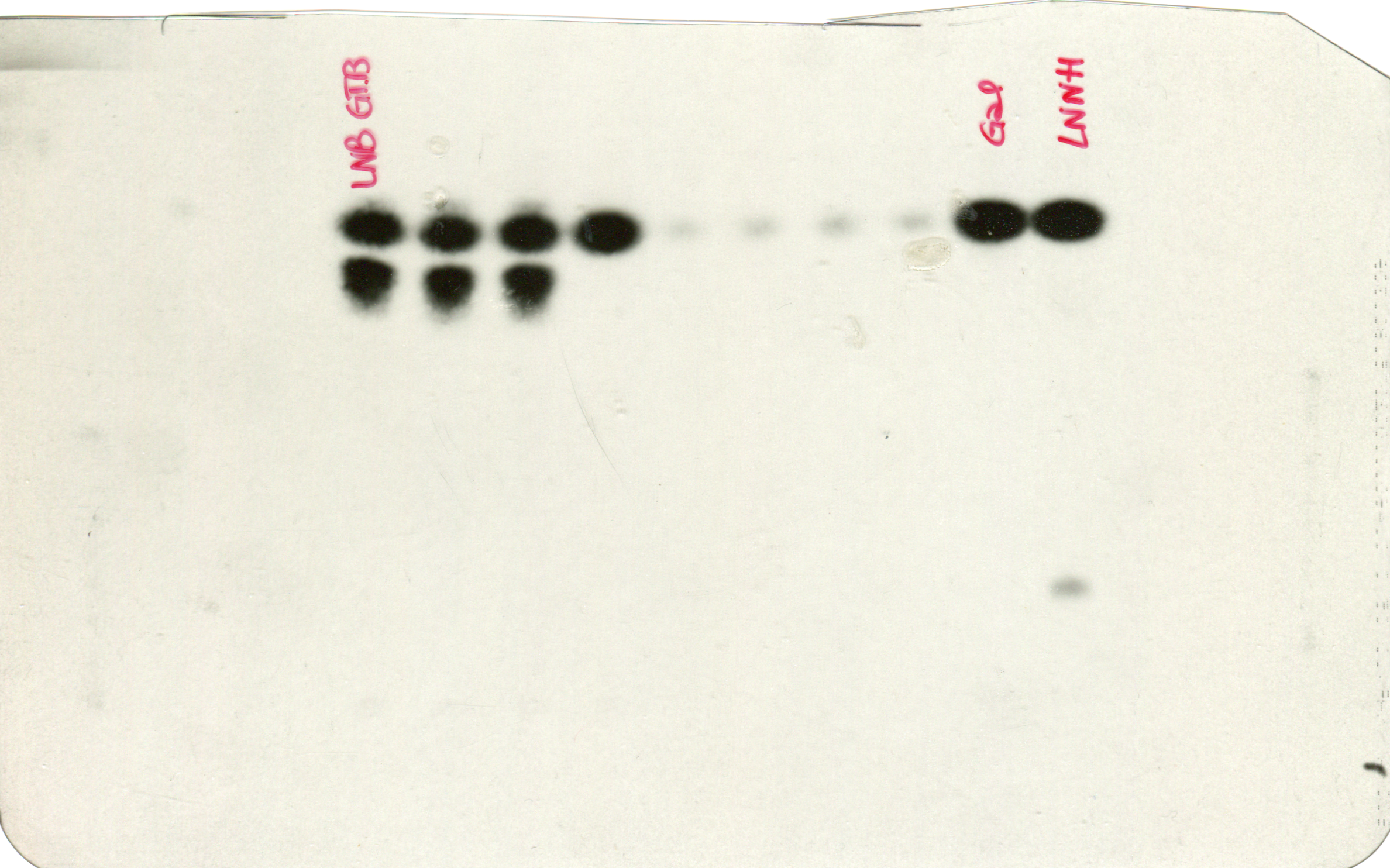

Supplement: Source data 1. [file elife-70272-supp3.zip › Source data 1/Figure 2 lanes 5-10 and Figure 2 supplement 2 source data.tif]

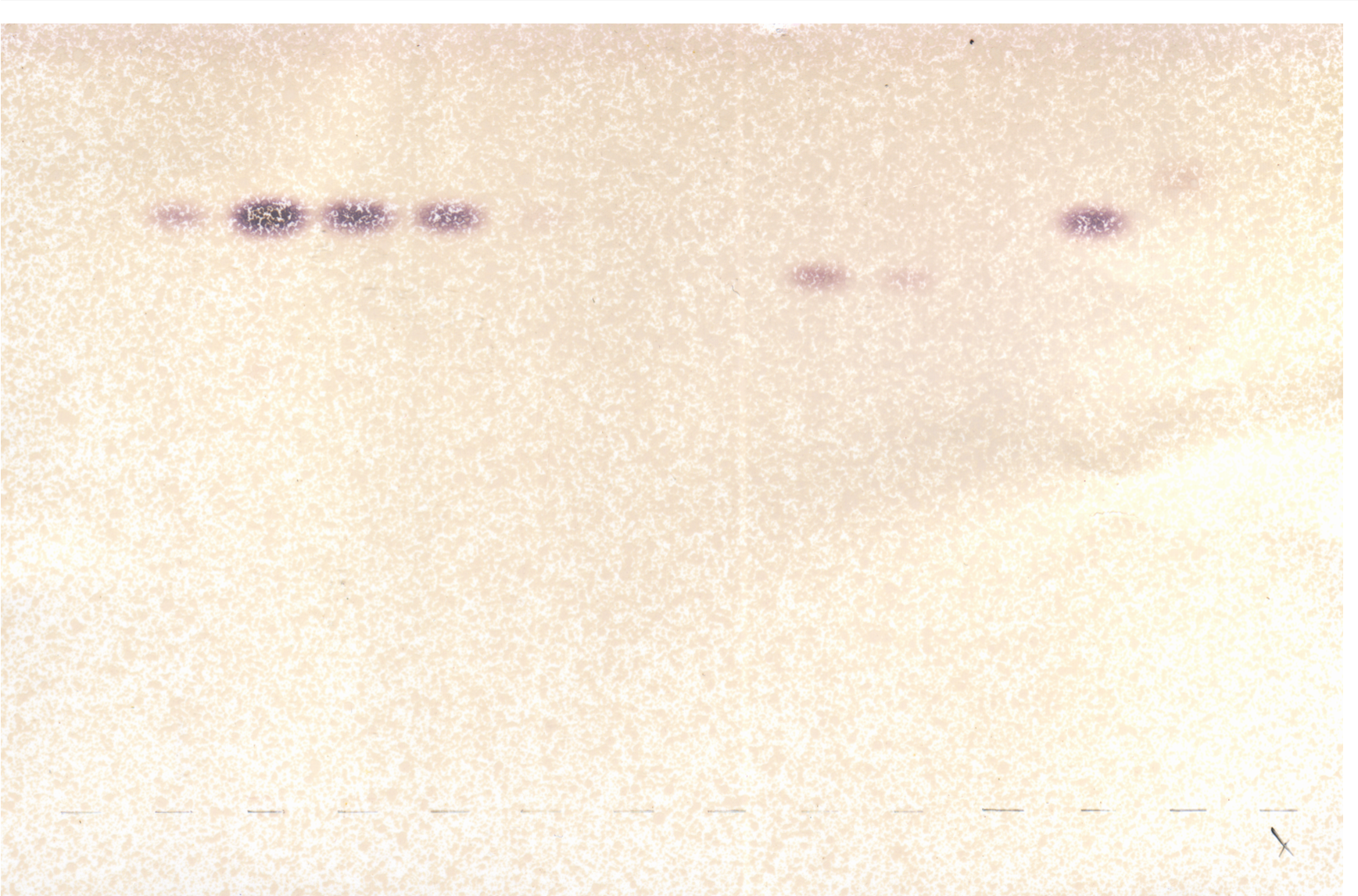

Supplement: Source data 1. [file elife-70272-supp3.zip › Source data 1/Figure 3 - figure supplement 2 source data 1.tiff]

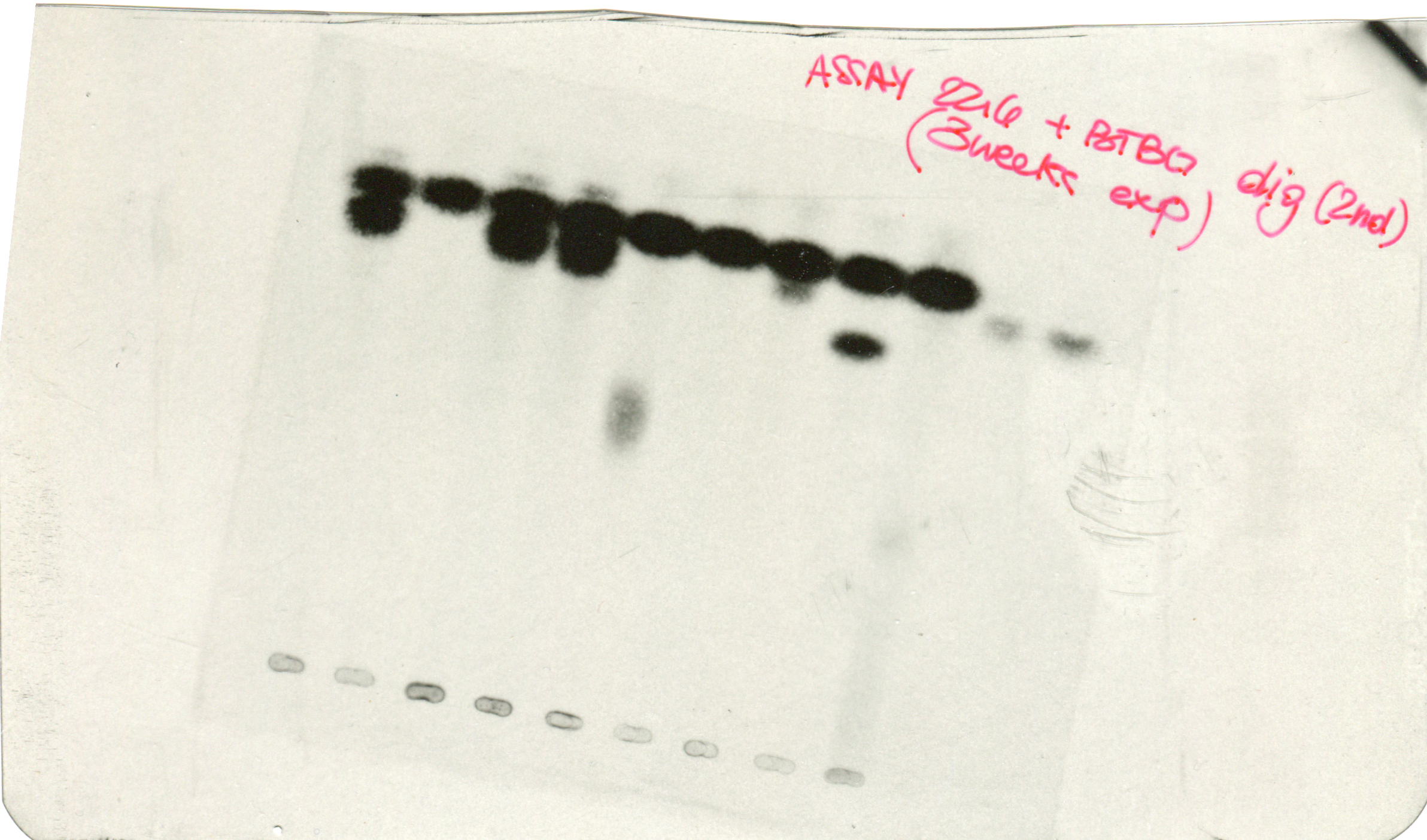

Supplement: Source data 1. [file elife-70272-supp3.zip › Source data 1/Figure 2 lane 20 source data.tif]

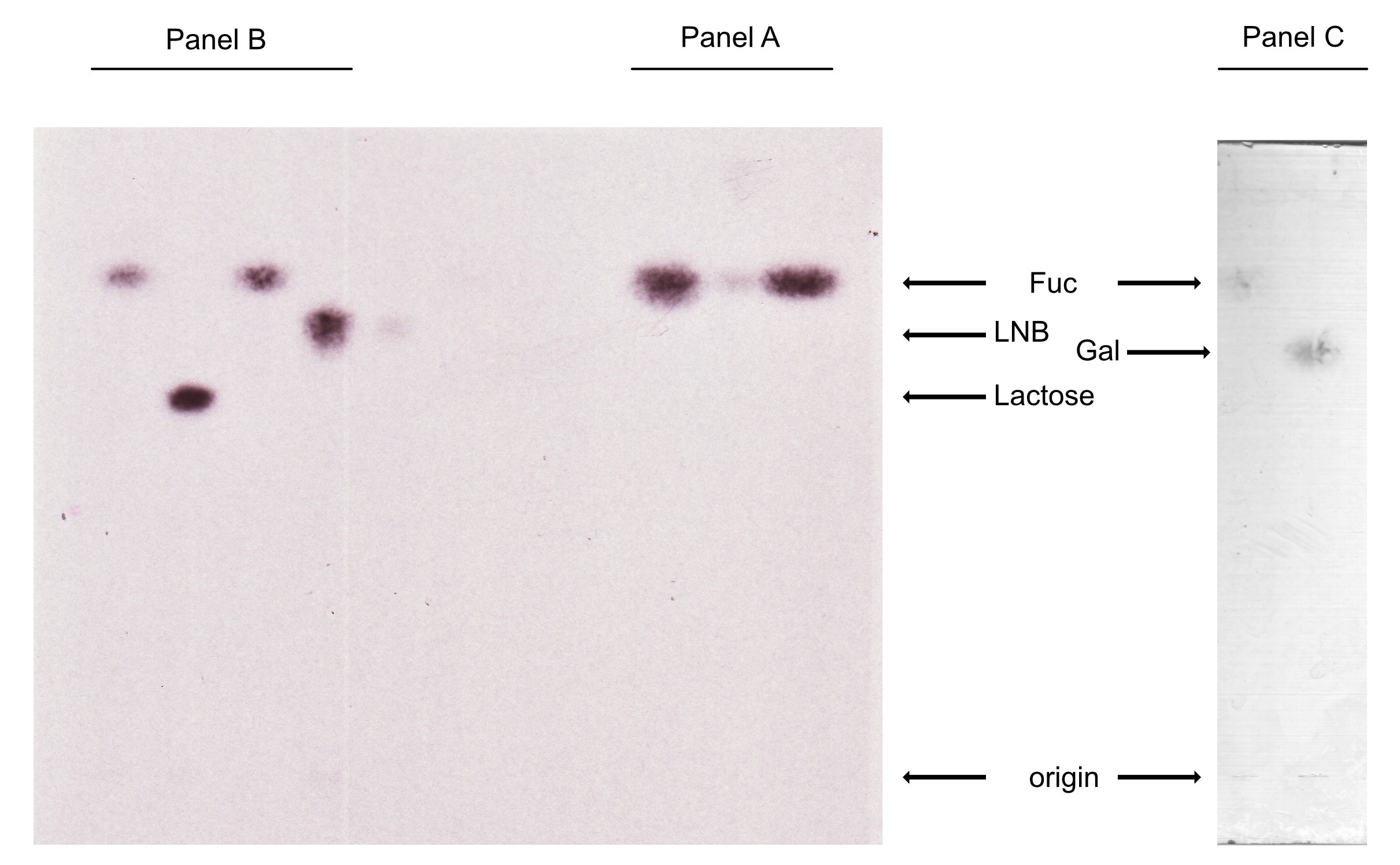

Supplement: Source data 1. [file elife-70272-supp3.zip › Source data 1/Figure 3 - figure supplement 1 source data.tiff]

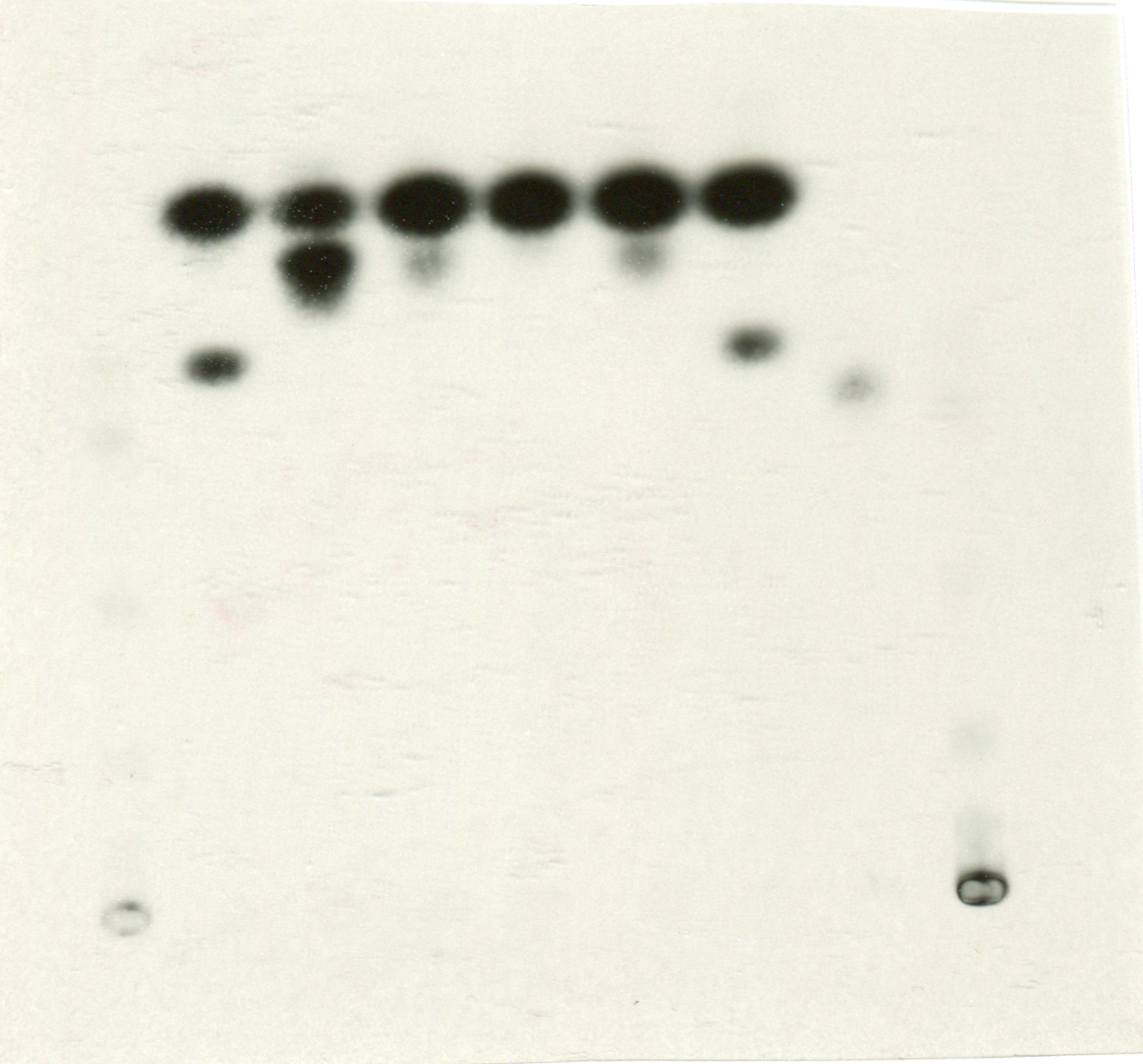

Supplement: Source data 1. [file elife-70272-supp3.zip › Source data 1/Figure 2 lanes 1-4 source data.tif]

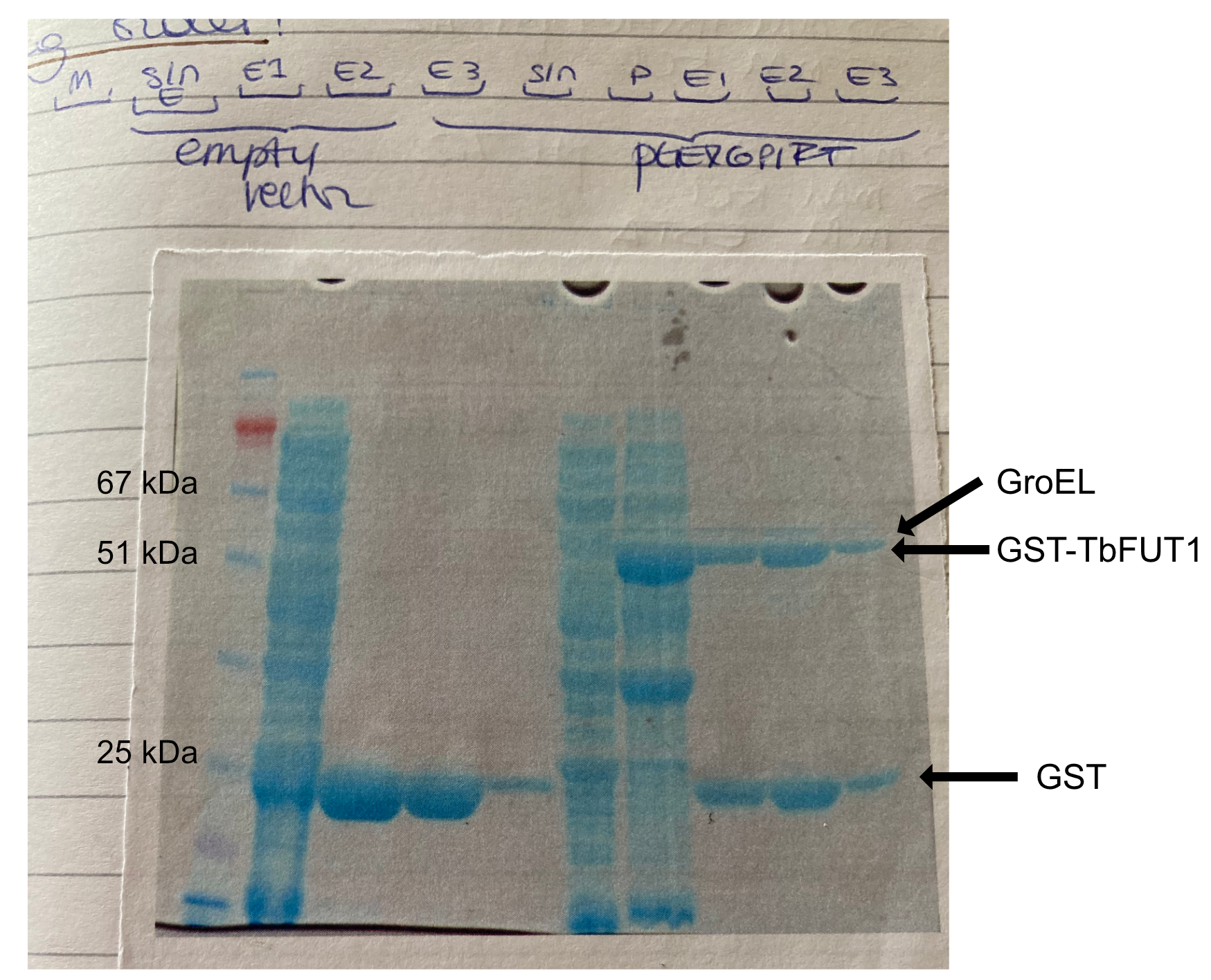

Supplement: Source data 1. [file elife-70272-supp3.zip › Source data 1/Figure 2 - figure supplement 1 source data 2.tiff]

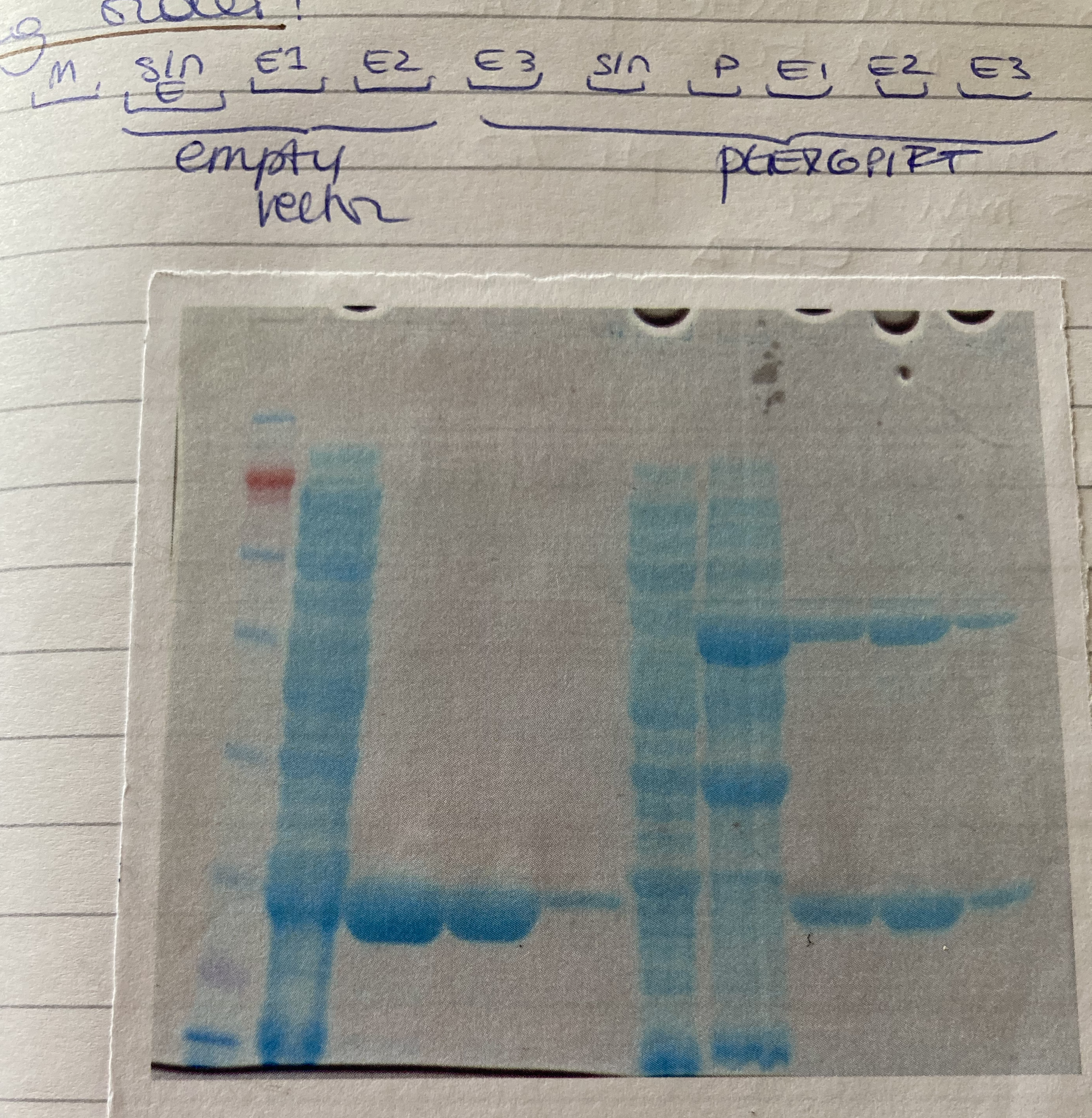

Supplement: Source data 1. [file elife-70272-supp3.zip › Source data 1/Figure 2 - figure supplement 1 source data.tif]

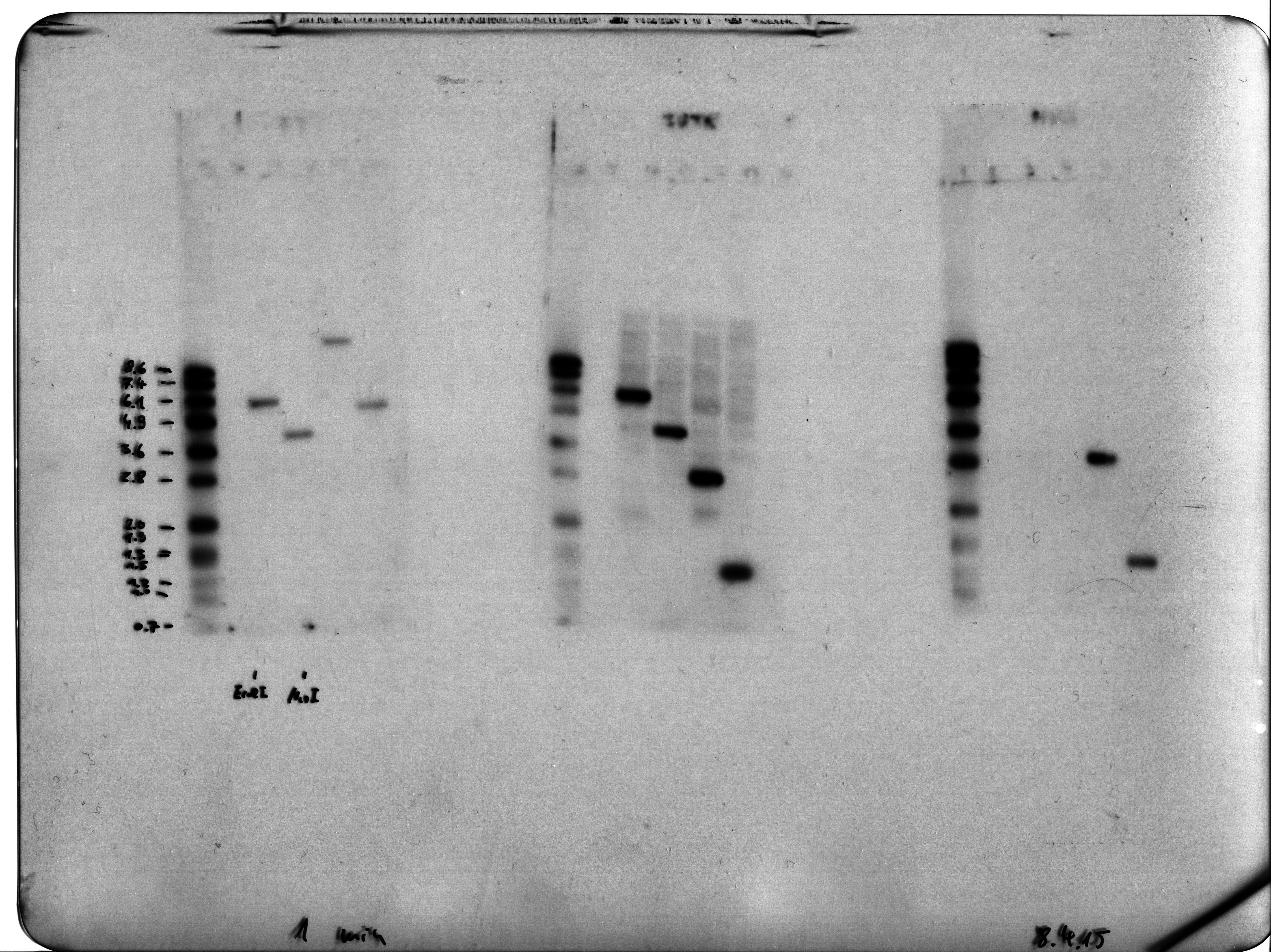

Supplement: Source data 2. [file elife-70272-supp4.zip › source data 2/Figure 5 - figure supplement 1 panels A and C source data.tif]

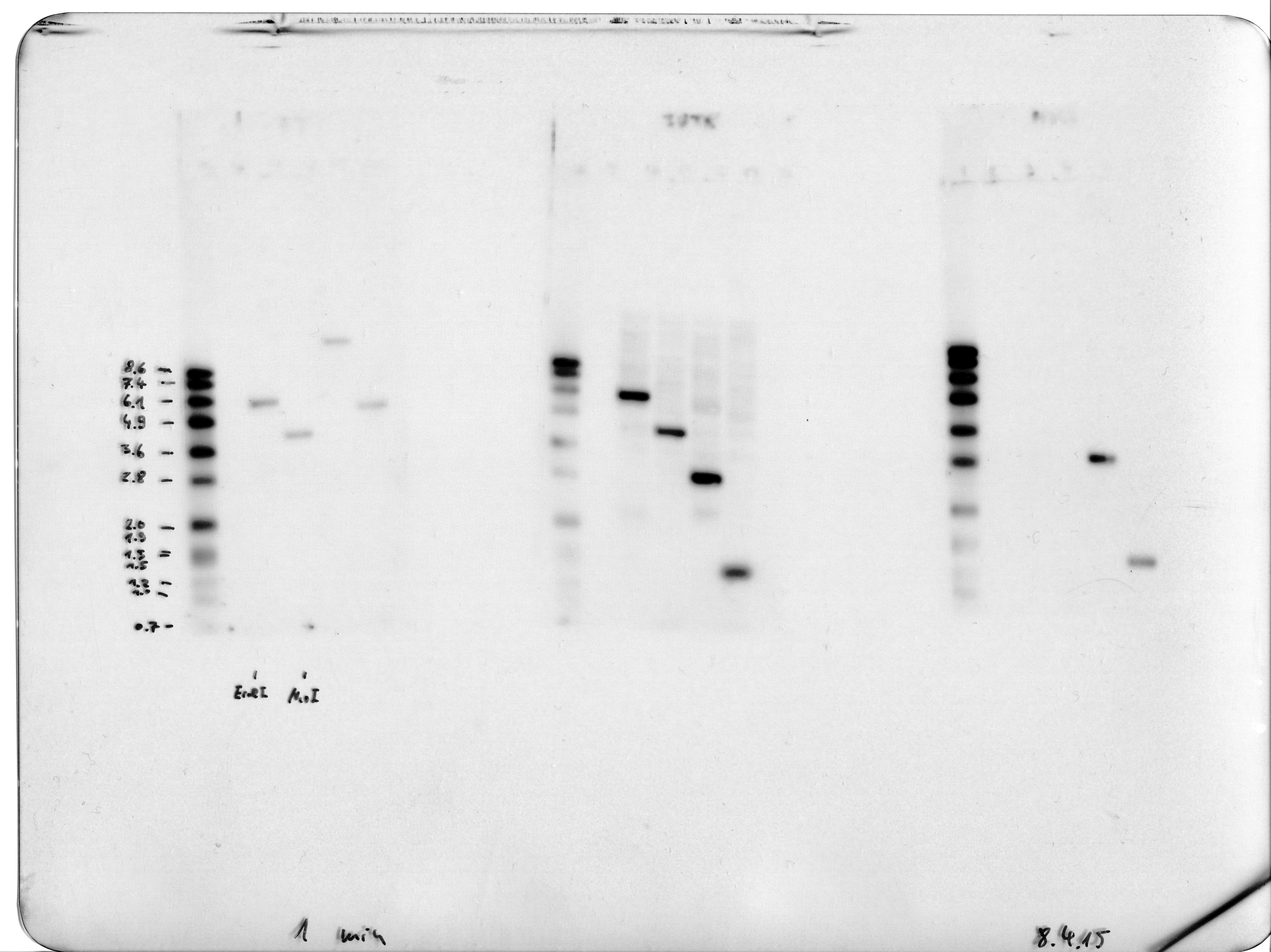

Supplement: Source data 2. [file elife-70272-supp4.zip › source data 2/Figure 5 - figure supplement 1 panel B source data.tif]

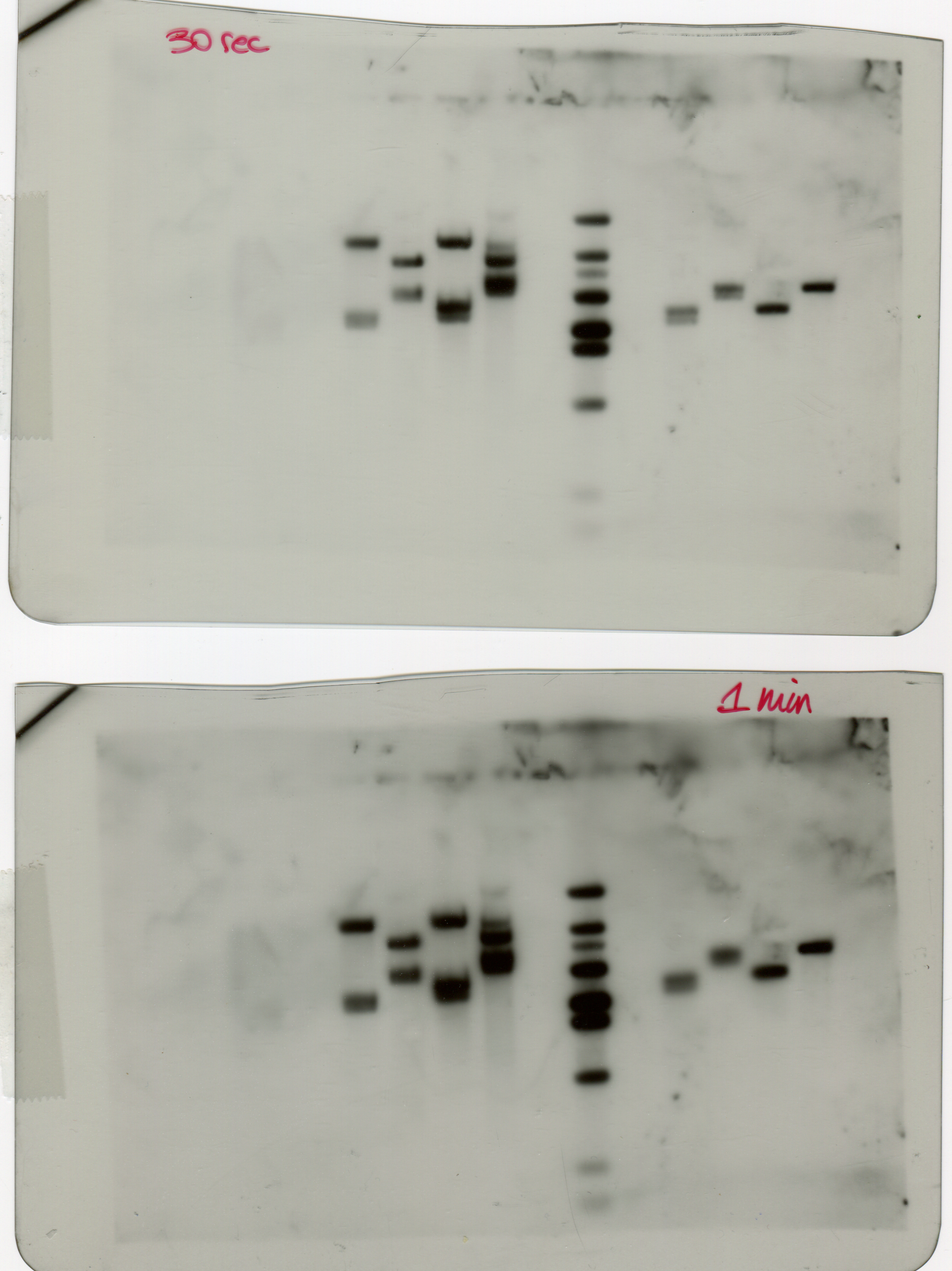

Supplement: Source data 2. [file elife-70272-supp4.zip › source data 2/Figure 5 - figure supplement 1 panel E source data.tif]

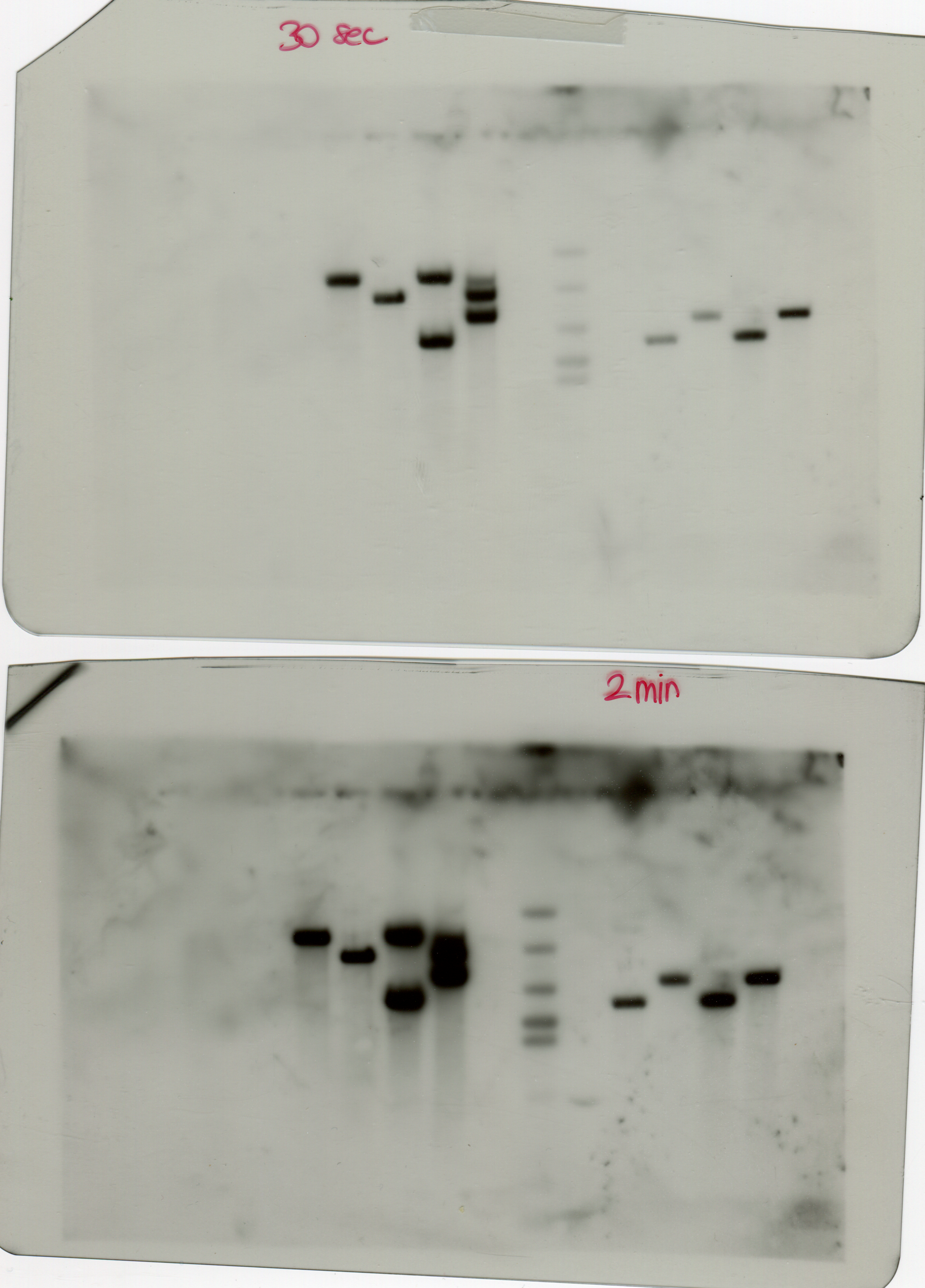

Supplement: Source data 2. [file elife-70272-supp4.zip › source data 2/Figure 5 - figure supplement 1 panel D source data.tif]

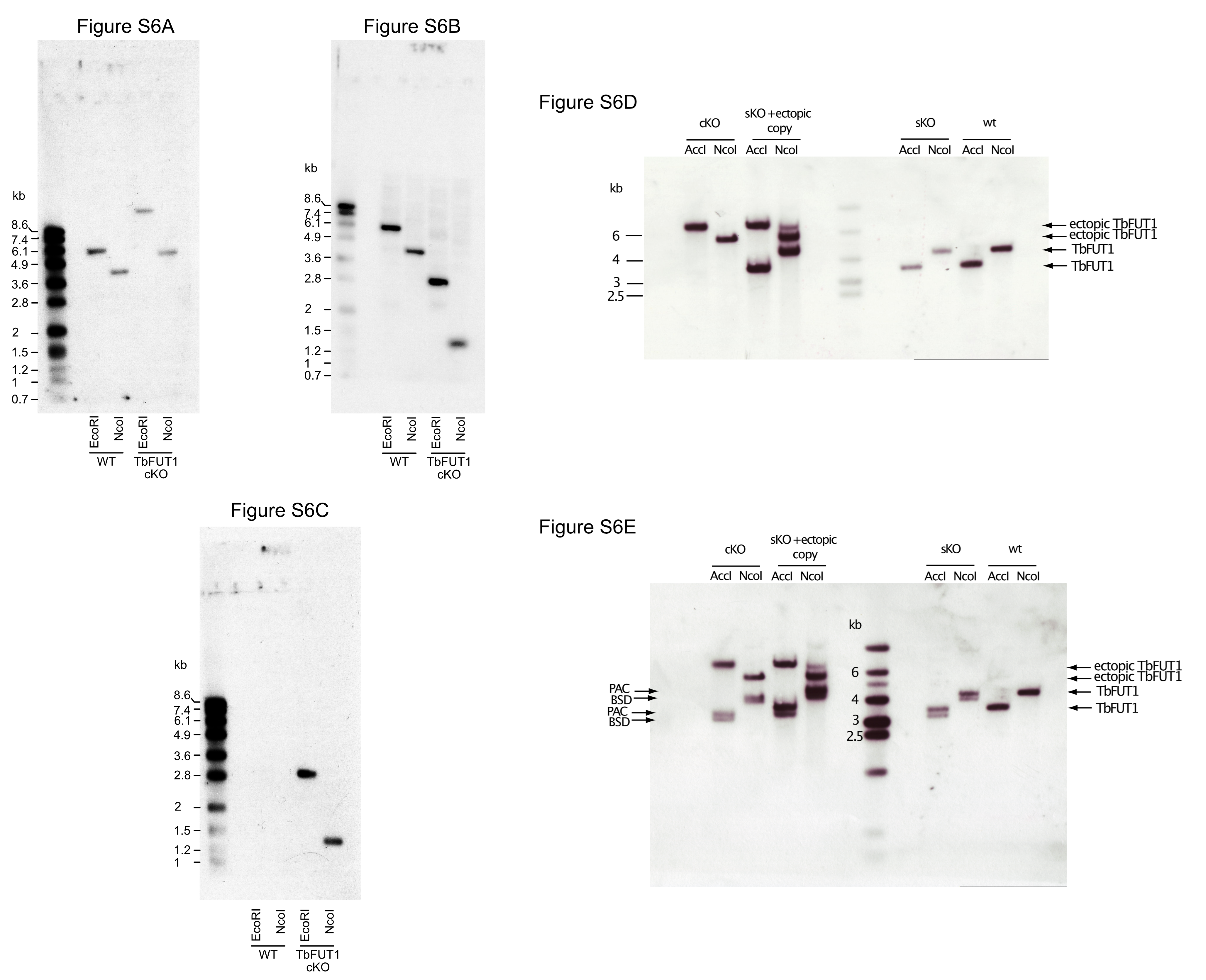

Supplement: Source data 2. [file elife-70272-supp4.zip › source data 2/Figure 5 - figure supplement 1 source data.tiff]

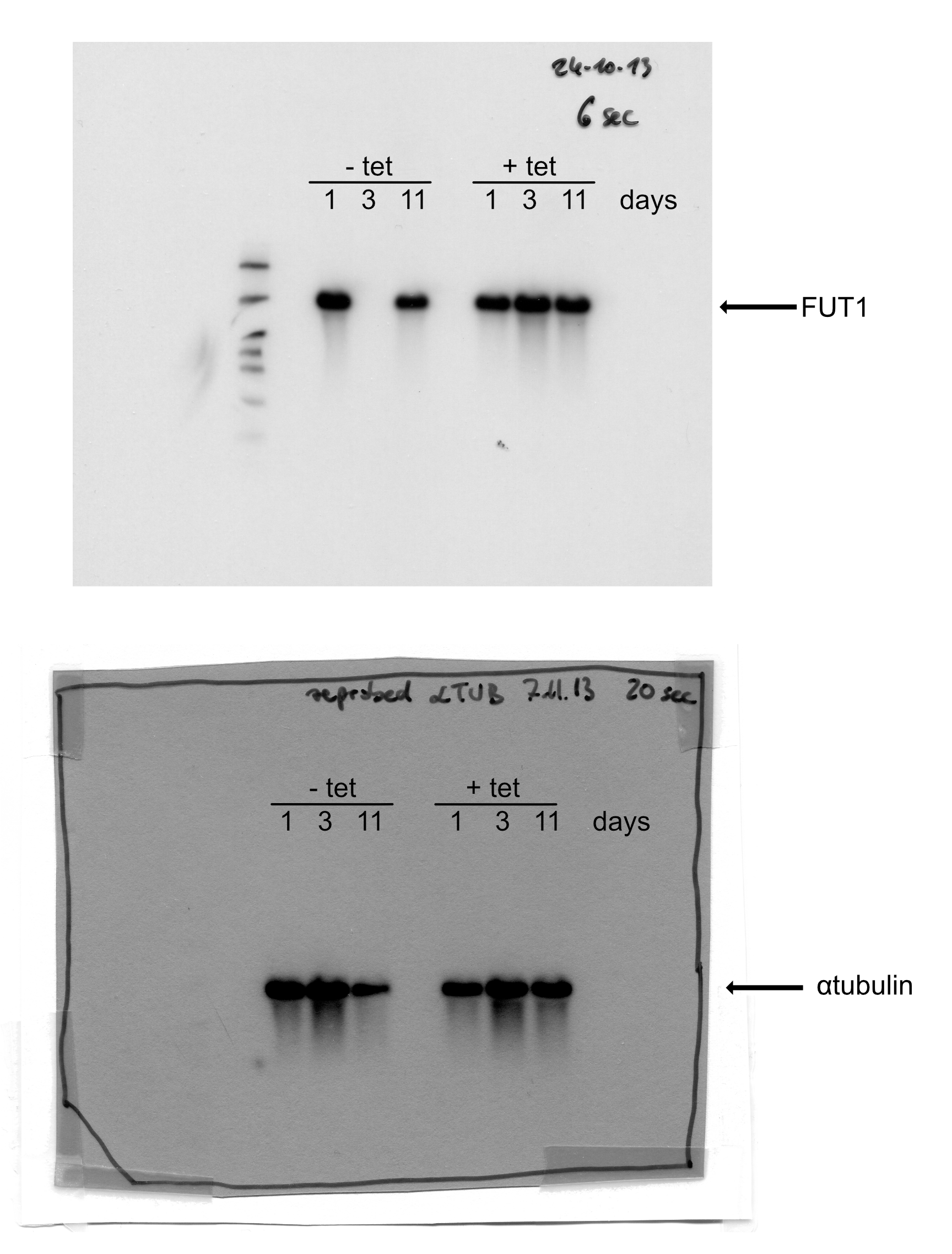

Supplement: Source data 3. [file elife-70272-supp5.zip › Source data 3/Figure 6E - source data 3.tiff]

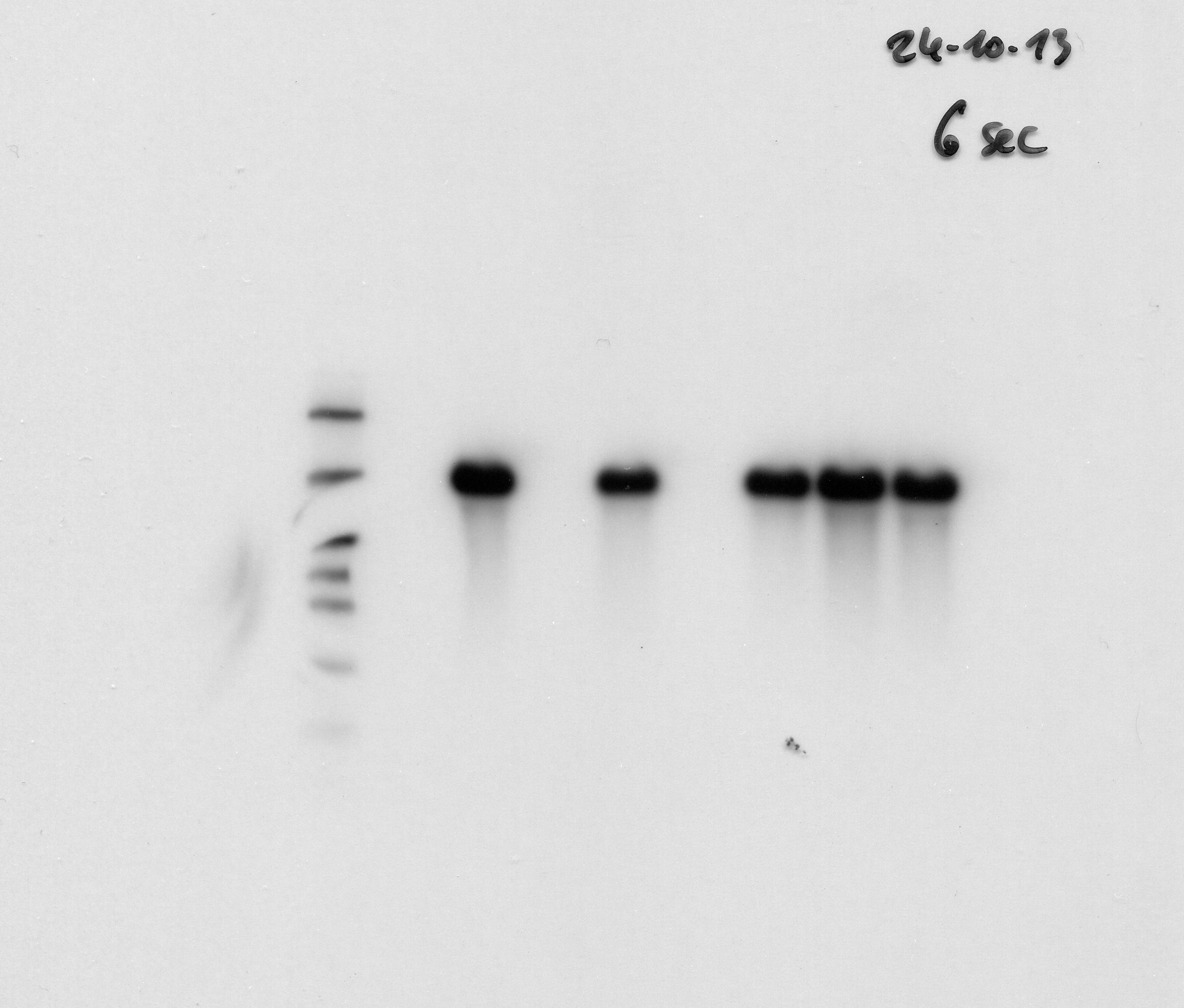

Supplement: Source data 3. [file elife-70272-supp5.zip › Source data 3/Figure 6E - source data 1.tif]

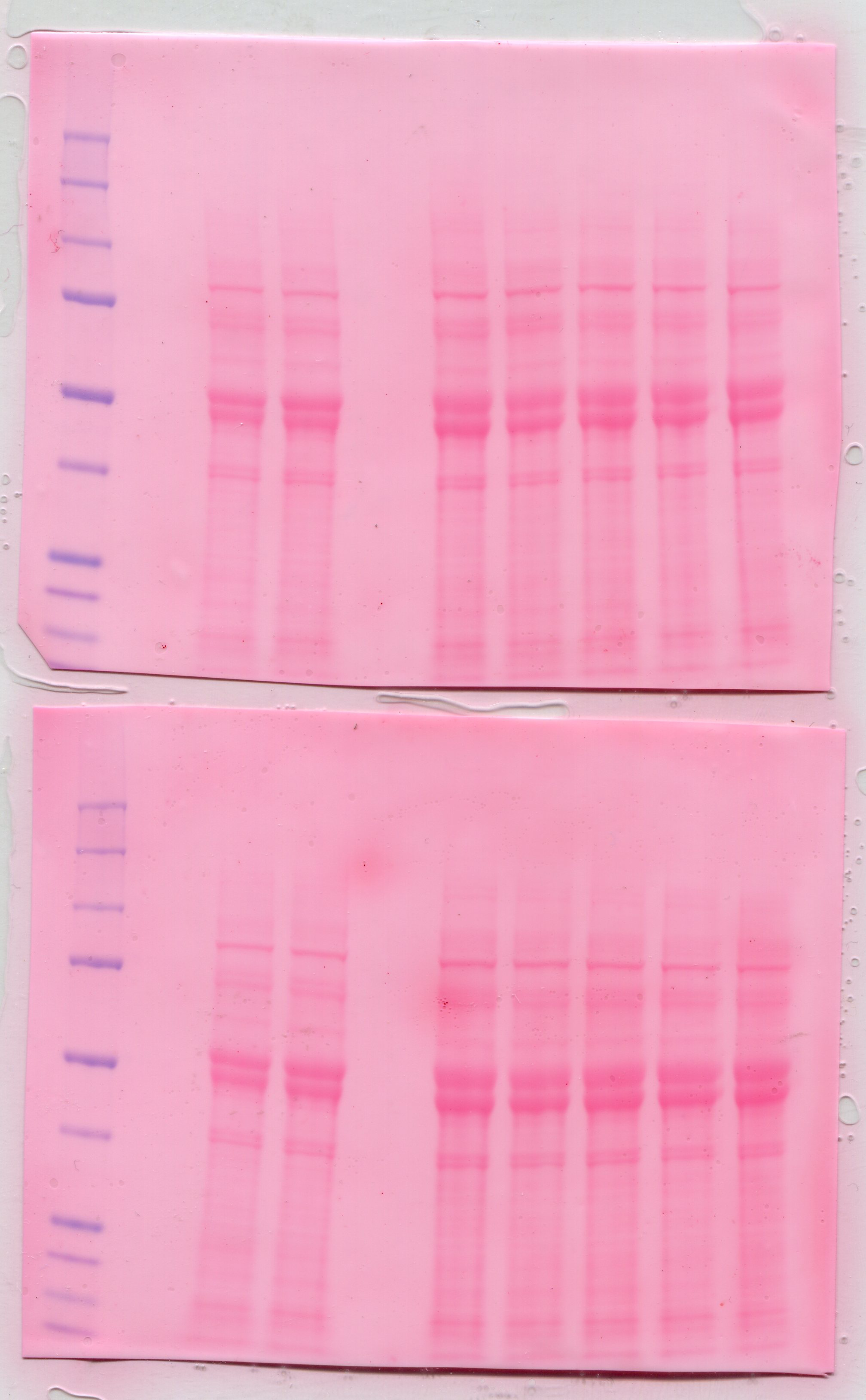

Supplement: Source data 3. [file elife-70272-supp5.zip › Source data 3/Figure 6F - source data 3.tif]

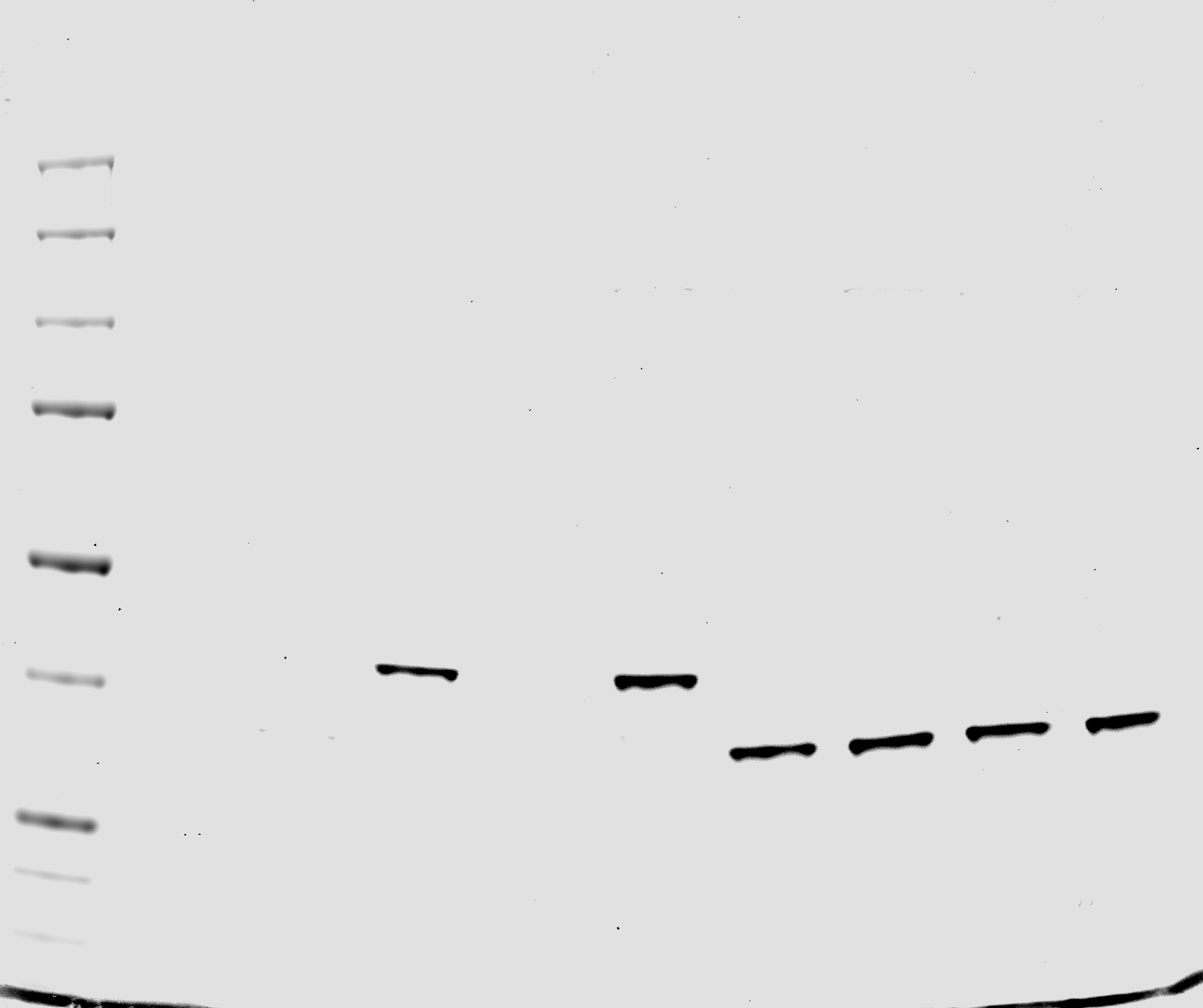

Supplement: Source data 3. [file elife-70272-supp5.zip › Source data 3/Figure 6F - source data 2.tif]

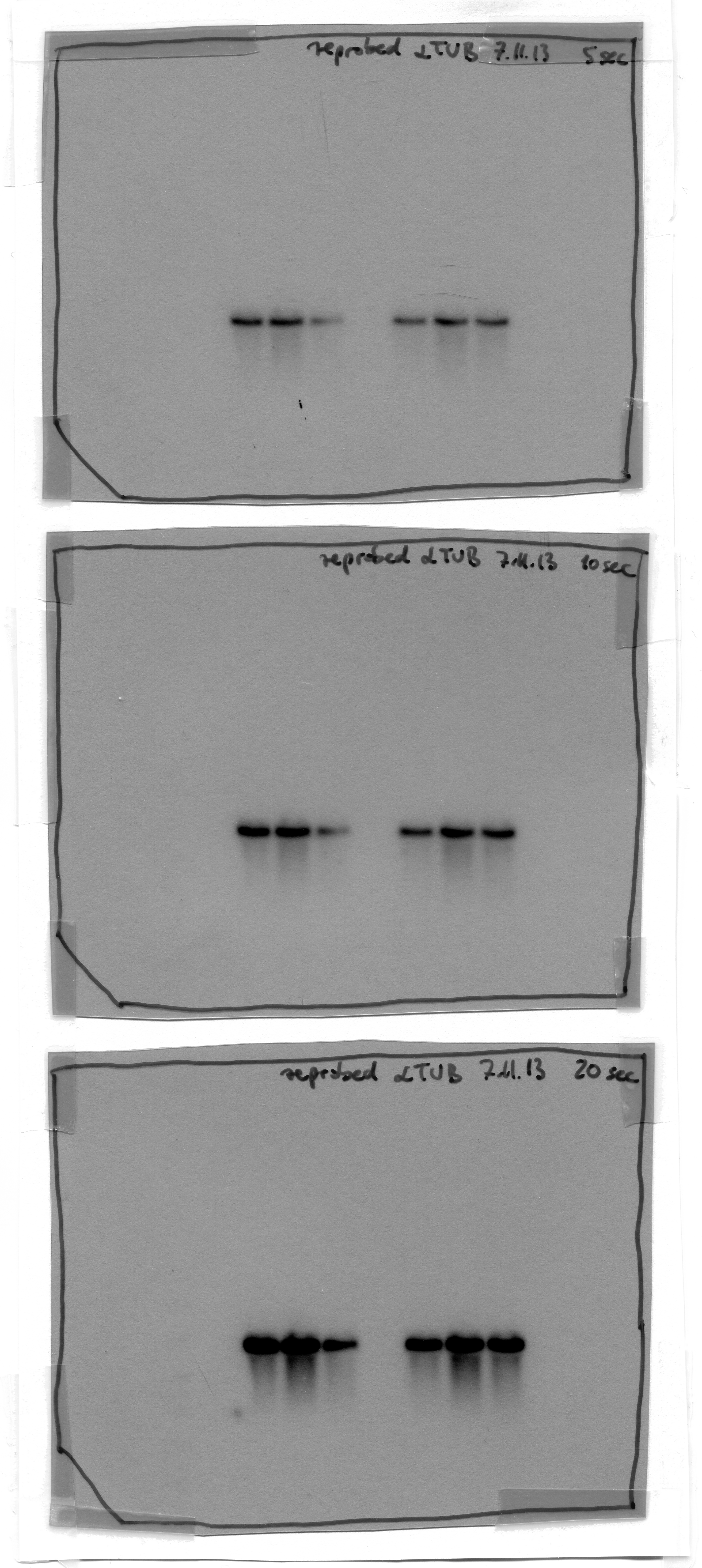

Supplement: Source data 3. [file elife-70272-supp5.zip › Source data 3/Figure 6E - source data 2.tif]

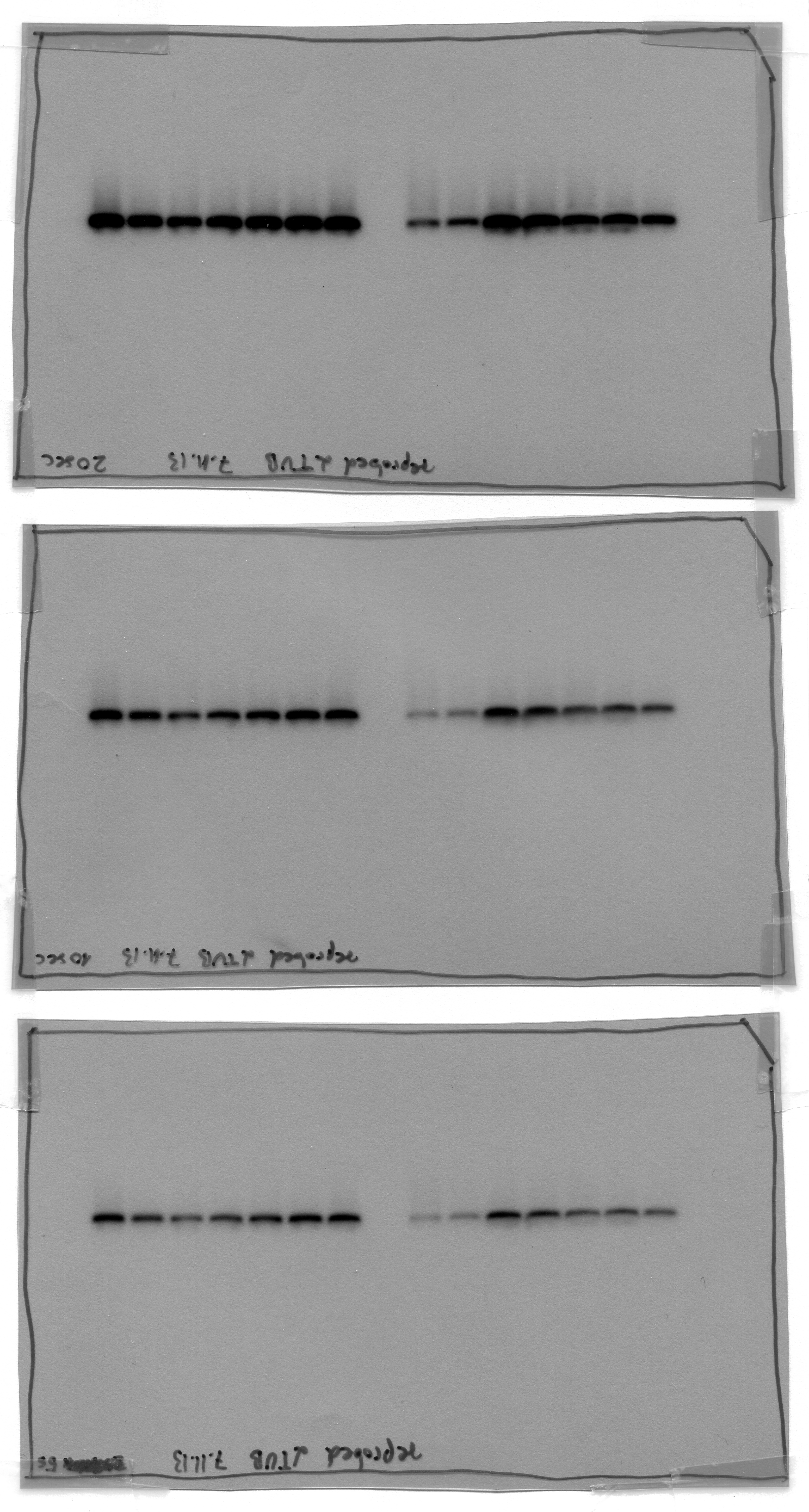

Supplement: Source data 3. [file elife-70272-supp5.zip › Source data 3/Figure 6D - source data 2.tif]

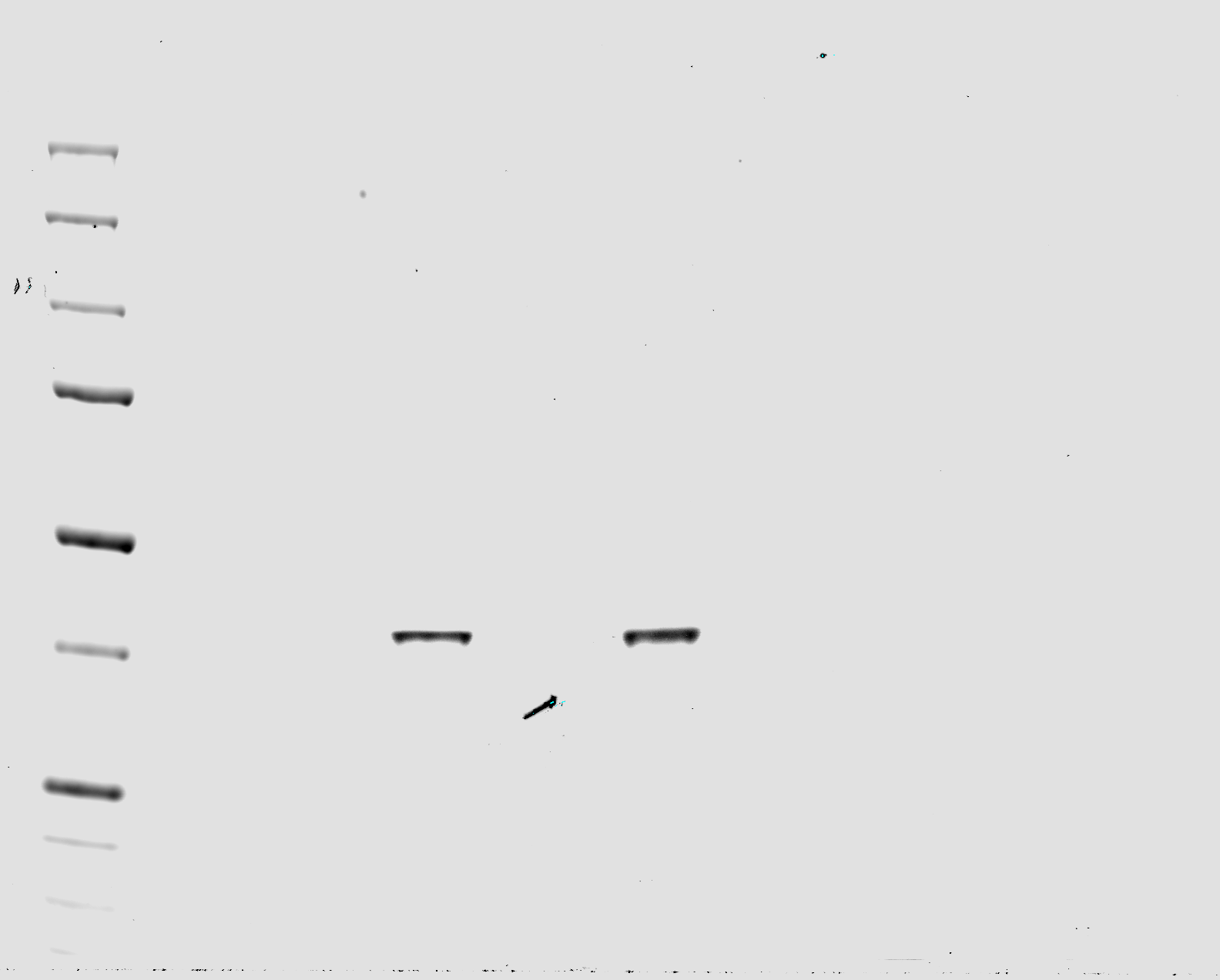

Supplement: Source data 3. [file elife-70272-supp5.zip › Source data 3/Figure 6F - source data 1.tif]

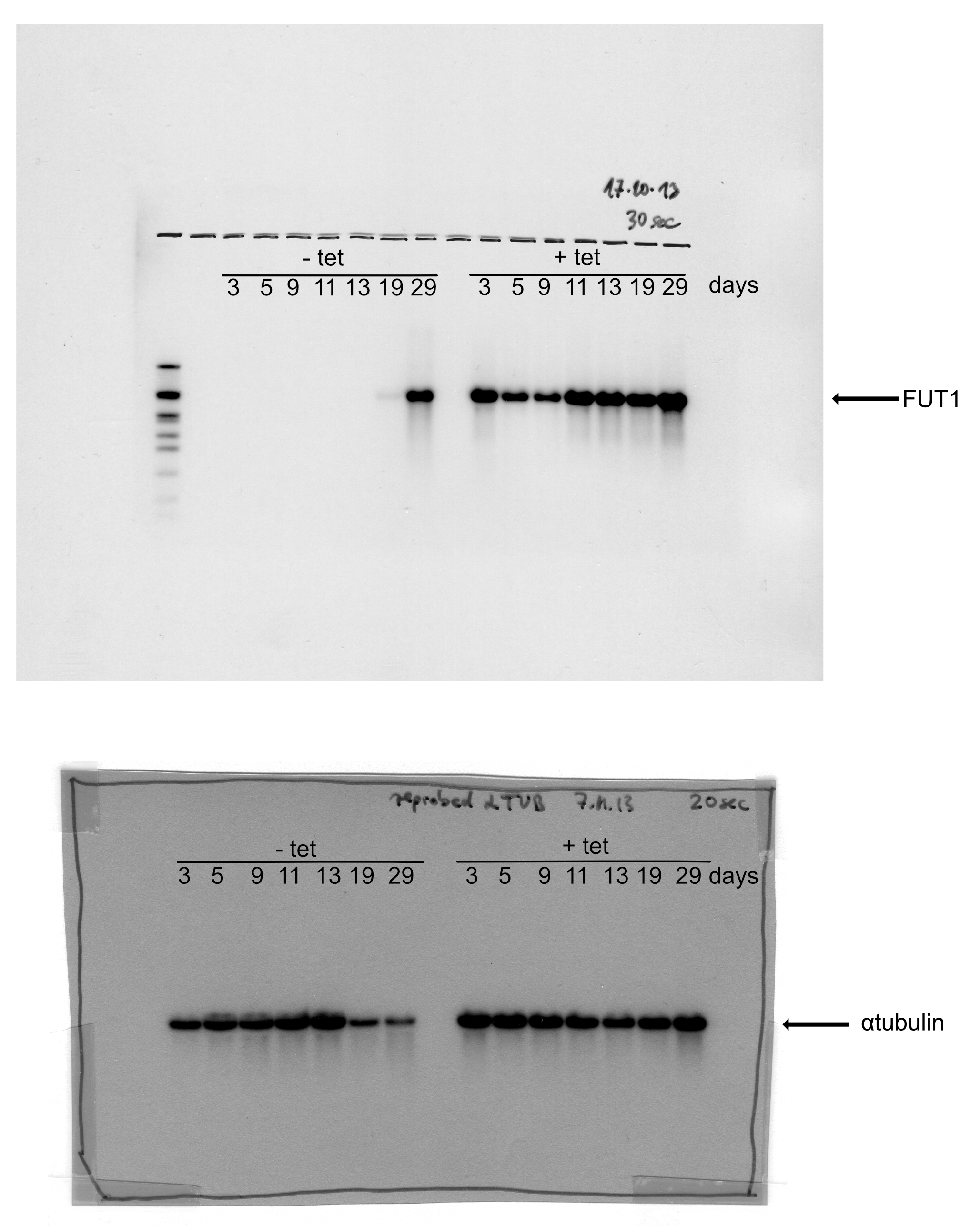

Supplement: Source data 3. [file elife-70272-supp5.zip › Source data 3/Figure 6D- source data 3.tiff]

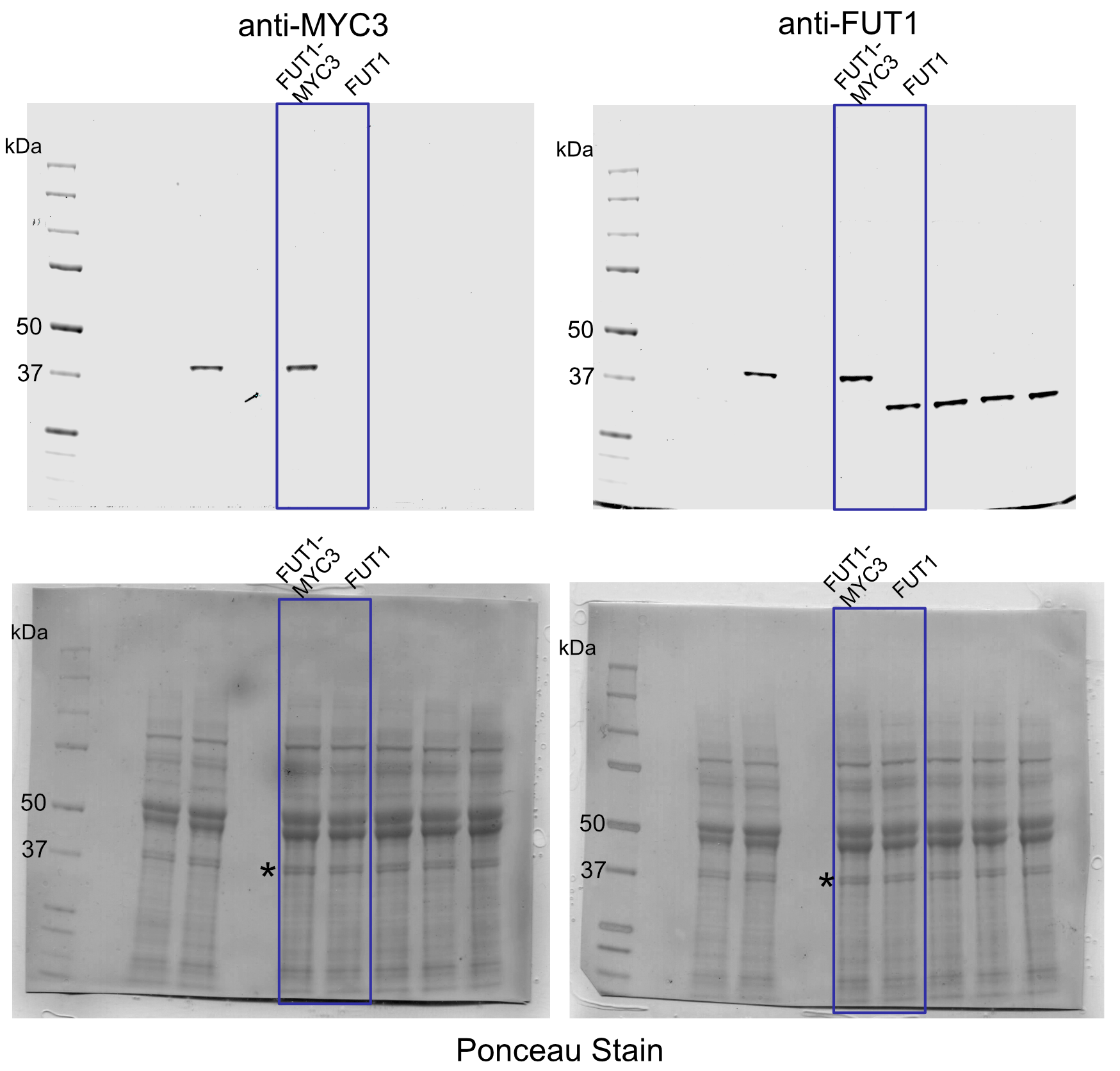

Supplement: Source data 3. [file elife-70272-supp5.zip › Source data 3/Figure 6F - source data 4.tiff]

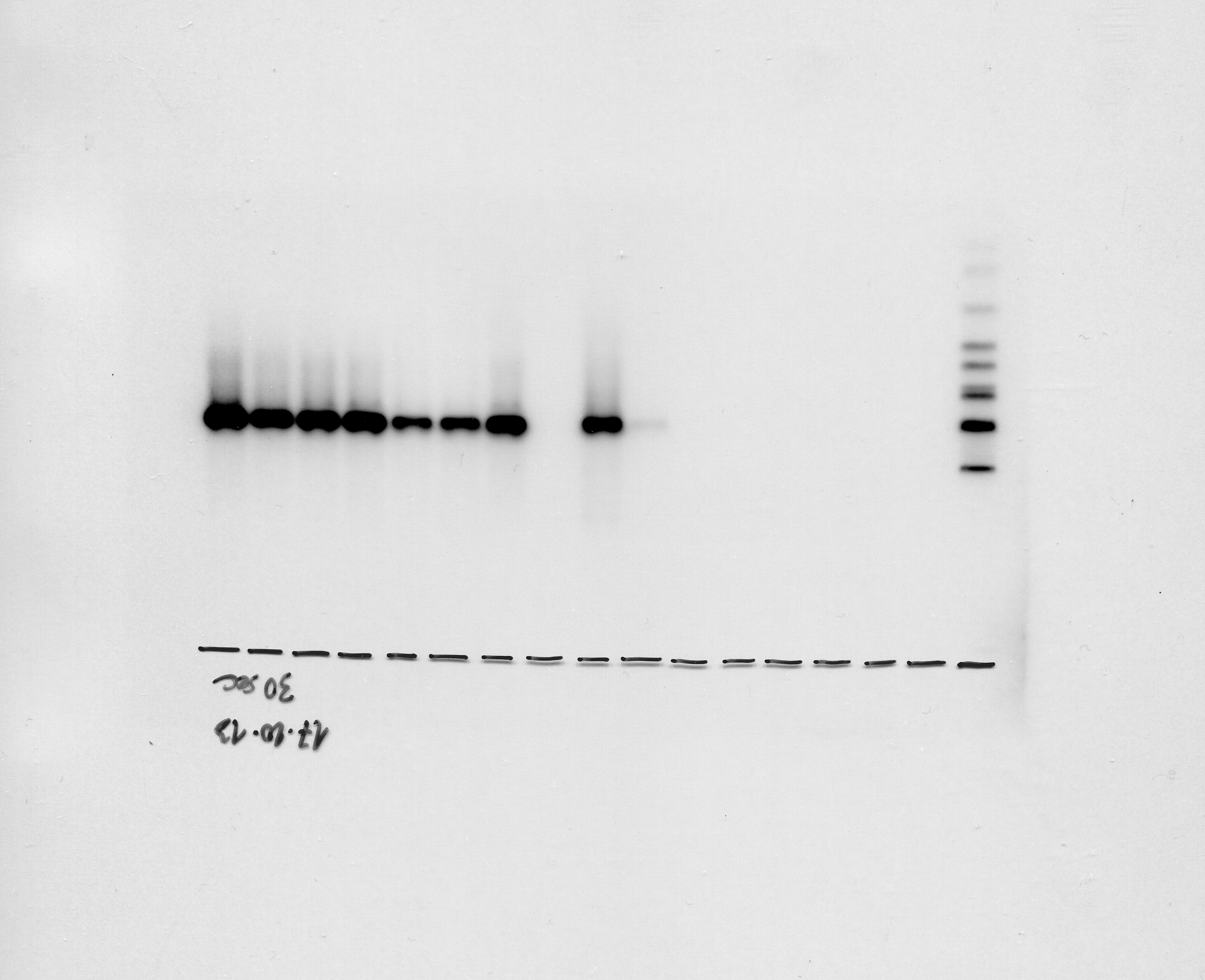

Supplement: Source data 3. [file elife-70272-supp5.zip › Source data 3/Figure 6D - source data.tif]
